# Shared Epitope Alleles Remain A Risk Factor for Anti-Citrullinated Proteins Antibody (ACPA) – Positive Rheumatoid Arthritis in Three Asian Ethnic Groups

**DOI:** 10.1371/journal.pone.0021069

**Published:** 2011-06-15

**Authors:** Too Chun-Lai, Leonid Padyukov, Jasbir Singh Dhaliwal, Emeli Lundström, Abqariyah Yahya, Nor Asiah Muhamad, Lars Klareskog, Lars Alfredsson, Per Tobias Larsson, Shahnaz Murad, for the Malaysian Epidemiological Investigation of Rheumatoid Arthritis (MyEIRA) Study Group

**Affiliations:** 1 Rheumatology Unit, Department of Medicine, Karolinska Institutet, Stockholm, Sweden; 2 Allergy and Immunology Research Center, Institute for Medical Research, Kuala Lumpur, Malaysia; 3 Institute of Environmental Medicine, Karolinska Institutet, Stockholm, Sweden; 4 Epidemiology and Biostatistics Unit, Institute for Medical Research, Kuala Lumpur, Malaysia; 5 Institute for Medical Research, Kuala Lumpur, Malaysia; National Institute of Dental and Craniofacial Research, United States of America

## Abstract

**Background:**

To investigate the associations between HLA-DRB1 shared epitope (SE) alleles and rheumatoid arthritis in subsets of rheumatoid arthritis defined by autoantibodies in three Asian populations from Malaysia.

**Methods:**

1,079 rheumatoid arthritis patients and 1,470 healthy controls were included in the study. Levels of antibodies to citrullinated proteins (ACPA) and rheumatoid factors were assessed and the PCR-SSO method was used for HLA-DRB1 genotyping.

**Results:**

The proportion of ACPA positivity among Malay, Chinese and Indian rheumatoid arthritis patients were 62.9%, 65.2% and 68.6%, respectively. An increased frequency of SE alleles was observed in ACPA-positive rheumatoid arthritis among the three Asian ethnic groups. HLA-DRB1*10 was highly associated with rheumatoid arthritis susceptibility in these Asian populations. HLA-DRB1*0405 was significantly associated with susceptibility to rheumatoid arthritis in Malays and Chinese, but not in Indians. HLA-DRB1*01 did not show any independent effect as a risk factor for rheumatoid arthritis in this study and HLA-DRB1*1202 was protective in Malays and Chinese. There was no association between SE alleles and ACPA- negative rheumatoid arthritis in any of the three Asian ethnic groups.

**Conclusion:**

The HLA-DRB1 SE alleles increase the risk of ACPA-positive rheumatoid arthritis in all three Asian populations from Malaysia.

## Introduction

Our knowledge on disease pathology and risk assignment in rheumatoid arthritis is mainly based on studies of Caucasian populations [1]. Investigations on different ethnic groups are, however, relatively rare and have not followed the rapidly increasing understanding of the genetic heterogeneity of rheumatoid arthritis (RA). There is thus a need for extended studies of genetic as well as environmental risk factors in different ethnic populations in order to achieve a deeper understanding of which different molecular pathways that are involved in the pathogenesis of different subsets of RA in different human populations.

Epidemiologic studies have demonstrated that RA is relatively common in almost all populations of the world, albeit a somewhat higher prevalence has been reported in Europe and the United States as compared to Asia [2]. Detailed studies on interactions between genetic and environmental risk factors for RA so far mainly been reported from Europe and the USA, and have demonstrated that the major genetic risk factor for RA, i.e. presence of certain HLA-DRB1 (MIM ID*142857) alleles exert their effects only in the subset of RA that is defined by the presence of ACPA [3], [4].

Human leukocyte antigens (HLA) account for one third to one half of the total genetic contribution to RA susceptibility [5], [6]. DRB1*0401, *0404 and *0408 are associated with RA in Caucasians, while the disease is mainly associated with DRB1*0405 in East Asians [7], [8], [9], [10], [11], [12], [13], [14]. In other ethnic groups, it has been well established that alternative HLA-DRB1 alleles might be associated with RA, either instead of or in addition to DRB1*04.

It was recently shown that HLA-DRB1 SE alleles are associated with increased levels of anti-citrullinated protein antibodies (ACPA) in Caucasian RA patients [15], [16], [17], [18]. It is unclear, however, whether this selective association between ACPA-positive RA and certain HLA-DRB1 alleles is valid in all ethnic groups, in particular in groups with distribution of HLA-DRB1 alleles different from those described in Caucasians. Few studies examining the relationship between HLA-DRB1 alleles and subsets of RA have been published from other Asian populations with slightly different results [10], [19], [20]. For example the DRB1 SE alleles were associated with both ACPA-positive RA cases (OR = 5.18) and ACPA-negative RA cases (OR = 2.31) in a Korean study, while no association between the DRB1 SE alleles and subsets of RA was observed in the Chinese patients from China (OR = 0.52) [10], [20].

Malaysia is a multiethnic country representing the genetic diversity across multiple large populations i.e. Malay, Chinese and Indian. In the present study, we determined the frequencies of the HLA-DRB1-shared epitope alleles and investigated their association with anti-citrullinated protein autoantibody-positive and –negative RA in the three Asian populations from Malaysia.

## Results

### Presence of ACPA in Malaysian RA populations

Stratification analysis by ethnic groups showed comparable prevalence of ACPA positivity in RA cases and controls. The overall prevalence of ACPA positivity was 66.0% in RA cases and 3.3% in the controls. The SE-positivity was noted to be significantly associated with RA in all the three ethnic groups (Malay OR = 3.98, 95%CI 2.94–5.40, p = 2.74×10^−20^; Chinese OR = 4.52, 95%CI 2.53–8.06, p = 7.69×10^−8^ and Indian OR = 2.16, 95% CI 1.49–3.12, p = 4.09×10^−5^) (Table 1).

**Table 1:** Baseline demographic characteristics of cases of rheumatoid arthritis (RA) and controls in MyEIRA, by ethnicity.

Characteristic	Malay		Chinese		Indian	
	RA	Controls	RA	Controls	RA	Controls
Female: Male n, (ratio)	386∶64 (6∶1)	809∶105 (8∶1)	187∶36 (5∶1)	172∶25 (7∶1)	290∶41 (7∶1)	232∶39 (6∶1)
Age, mean (SD) years	46.2 (11.8)	46.3 (11.3)	52.6 (11.2)	50.9 (11.5)	47.7 (10.9)	48.1 (10.6)
IgM RF positive (%)	204 (47.8)	31(4.0)	103(48.1)	6 (3.2)	172(55.1)	12 (4.9)
IgG RF positive (% )	213 (49.8)	58 (7.4)	105 (49.1)	13 (7.1)	167 (53.5)	16 (6.5)
ACPA-positive (% )	278 (62.9)	25(3.1)	144 (65.2)	7 (3.8)	223 (68.6)	9 (3.5)
SE* positive (% )	146(36.0)	85(12.4)	70(34.3)	17(11.6)	143 (48.3)	65 (30.5)

RF: rheumatoid factor, ACPA: anti-citrullinated proteins antibody, SE: shared epitope.

*patients and controls carrying one or two alleles of shared epitope were classified as SE-positive.

The percentages calculated in each characteristic category were based on the number of available data.

### Distribution of HLA-DRB1 genotype and risk of developing ACPA-positive and ACPA-negative RA

In order to investigate the influence of HLA-DRB1 alleles with regard to the risk of developing ACPA-positive RA or ACPA-negative RA, we performed analyses stratified on ACPA status among the cases. The frequencies of HLA-DRB1 alleles were different for several DRB1 alleles among ACPA-positive and ACPA-negative RA patients in comparison with ethnically matched controls as demonstrated in Table 2, Table 3 and Table 4 for Malay, Chinese and Indian ethnic groups, respectively.

**Table 2:** Carrier frequencies of HLA-DRB1in the Malay ethnic group by ACPA status.

	Malay			
	Cases (n = 405)(%)	Controls (n = 685)(%)	OR, 95% CI§	P value (Pc)
DRB1*01				
ACPA-positive	4 (2)	-	0.97(0.31–3.08)	
ACPA-negative	4 (3)	11(2)	1.69(0.53–5.38)	
DRB1*03				
ACPA-positive	16 (6)	-	0.61(0.35–1.08)	
ACPA-negative	15(10)	67(10)	1.03(0.57–1.86)	
DRB1*04				
ACPA-positive	98(38)	-	4.10(2.93–5.73)	9×10^−18^ (1×10^−16^)
ACPA-negative	25 (17)	90(13)	1.33(0.82–2.16)	
DRB1*07				
ACPA-positive	31(12)	-	0.54(0.36–0.82)	
ACPA-negative	16(10)	139(20)	0.47(0.27–0.82)	0.0065(NS)
DRB1*08				
ACPA-positive	6(2)	-	0.43(0.18–1.04)	
ACPA-negative	12(8)	36(5)	1.58(0.80–3.11)	
DRB1*09				
ACPA-positive	17(7)	-	1.09(0.61–1.95)	
ACPA-negative	9(6)	42(6)	0.98(0.47–2.07)	
DRB1*10				
ACPA-positive	38(15)	-	6.11(3.45–10.82)	5×10^−12^ (6×10^−11^)
ACPA-negative	8(5)	19(3)	1.99(0.85–4.63)	
DRB1*11				
ACPA-positive	5(2)	-	0.27(0.11–0.69)	
ACPA-negative	11(7)	47(7)	1.08(0.55–2.14)	
DRB1*12				
ACPA-positive	118(46)	-	0.76(0.57–1.01)	
ACPA-negative	67(45)	363(53)	0.72(0.51–1.03)	
DRB1*13				
ACPA-positive	8(3)	-	0.44(0.20–0.94)	
ACPA-negative	10(7)	47(7)	0.98(0.48–1.98)	
DRB1*14				
ACPA-positive	14(6)	-	0.58(0.32–1.06)	
ACPA-negative	12(8)	62(9)	0.88(0.46–1.68)	
DRB1*15				
ACPA-positive	108(42)	-	0.83(0.62–1.10)	
ACPA-negative	79(53)	321(47)	1.28(0.90–1.83)	
DRB1*16				
ACPA-positive	29(11)	-	1.91(1.16–3.12)	0.0094(NS)
ACPA-negative	15(10)	43(6)	1.67(0.90–3.10)	

*The frequency of DRB1 genotypes in ACPA subgroups was calculated as the number of ACPA-positive or ACPA-negative RA cases for a specific DRB1 allele divided by the total number of cases within that ACPA subgroup (Malay: 256 ACPA-positive RA cases and 149 ACPA-negative RA cases).

§Individuals without the allele being investigated were used as the reference group for the calculation of odds ratio (OR) and 95% confidence interval (95% CI).

Corrected p (p_c_) values were obtained by multiplying the uncorrected p values by 13.

NS: not significant.

**Table 3:** Carrier frequencies of HLA-DRB1in the Chinese ethnic group by ACPA status.

	Chinese			
	Cases (n = 204)(%)	Controls (n = 164)(%)	OR, 95% CI§	P value (Pc)
DRB1*01				
ACPA-positive	2(2)	-	1.25(0.17–8.97)	
ACPA-negative	1(1)	2(1)	1.41(0.11–1.79)	
DRB1*03				
ACPA-positive	9(7)	-	0.36(0.16–0.78)	0.008(NS)
ACPA-negative	8(11)	28(17)	0.61(0.26–1.41)	
DRB1*04				
ACPA-positive	64(48)	-	5.23(3.03–9.03)	6×10^−10^ (7×10^−9^)
ACPA-negative	13(18)	25(15)	1.23(0.59–2.56)	
DRB1*07				
ACPA-positive	7(5)	-	0.86(0.32–2.33)	
ACPA-negative	0(0)	10(6.)	0.11(0.01–1.86)	
DRB1*08				
ACPA-positive	14(11)	-	0.77(0.38–1.56)	
ACPA-negative	14(19)	22(13)	1.56(0.75–3.25)	
DRB1*09				
ACPA-positive	41(31)	-	1.31(0.79–2.18)	
ACPA-negative	14(19)	42(26)	0.7(0.35–1.39)	
DRB1*10				
ACPA-positive	10(8)	-	13.36(1.69–105.78)	0.0016(0.0208)
ACPA-negative	4(6)	1(0.01)	9.59(1.05–87.36)	
DRB1*11				
ACPA-positive	11(8)	-	1.06(0.46–2.44)	
ACPA-negative	8(11)	13(8)	1.45(0.57–3.67)	
DRB1*12				
ACPA-positive	27(20)	-	0.66(0.38–1.14)	
ACPA-negative	19(26)	46(28)	0.92(0.49–1.72)	
DRB1*13				
ACPA-positive	4(3)	-	0.48(0.15–1.57)	
ACPA-negative	2(3)	10(6)	0.43(0.09–2.00)	
DRB1*14				
ACPA-positive	9(7)	-	0.3(0.14–0.66)	0.0016(0.0208)
ACPA-negative	18(25)	32(10)	1.38(0.71–2.66)	
DRB1*15				
ACPA-positive	37(28)	-	1.00(0.60–1.66)	
ACPA-negative	26(36)	46(28)	1.45(0.80–2.61)	
DRB1*16				
ACPA-positive	21(16)	-	1.00(0.54–1.88)	
ACPA-negative	10(14)	26(16)	0.86 (0.39–1.88)	

*The frequency of DRB1 genotypes in ACPA subgroups was calculated as the number of ACPA-positive or ACPA-negative RA cases for a specific DRB1 allele divided by the total number of cases within that ACPA subgroup (Chinese: 132 ACPA-positive RA cases and 72 ACPA-negative RA cases).

§Individuals without the allele being investigated were used as the reference group for the calculation of odds ratio (OR) and 95% confidence interval (95% CI).

Corrected p (p_c_) values were obtained by multiplying the uncorrected p values by 13.

NS: not significant.

**Table 4:** Carrier frequencies of HLA-DRB1in the Indian ethnic group by ACPA status.

	Indian			
	Cases (n = 296(%)	Controls (n = 213)(%)	OR, 95% CI§	P value (Pc)
DRB1*01				
ACPA-positive	12(6)	-	2.20(0.81–5.98)	
ACPA-negative	4(4)	6(3)	1.50(0.41–5.44)	
DRB1*03				
ACPA-positive	19(10)	-	1.01(0.52–1.96)	
ACPA-negative	15(19)	20(9)	1.79(0.87–3.66)	
DRB1*04				
ACPA-positive	69(35)	-	1.55(1.01–2.37)	NS (NS)
ACPA-negative	26(27)	54(25)	1.09(0.63–1.89)	
DRB1*07				
ACPA-positive	45(23)	-	0.79(0.51–1.25)	
ACPA-negative	20(21)	57(27)	0.72(0.40–1.28)	
DRB1*08				
ACPA-positive	12(6)	-	0.98(0.44–2.21)	
ACPA-negative	8(8)	13(6)	1.40(0.56–3.49)	
DRB1*09				
ACPA-positive	7(4)	-	3.83(0.79–18.64)	
ACPA-negative	2(2)	2(1)	2.24(0.31–16.18)	
DRB1*10				
ACPA-positive	75(38)	-	2.60(1.66–4.06)	2×10^−5^ (3×10^−4^)
ACPA-negative	21(22)	40(19)	1.21(0.67–2.19)	
DRB1*11				
ACPA-positive	10(5)	-	0.61(0.27–1.36)	
ACPA-negative	5(5)	17(8)	0.63(0.23–1.77)	
DRB1*12				
ACPA-positive	7(4)	-	0.42(0.17–1.03)	
ACPA-negative	9(9)	17(8)	1.19(0.51–2.78)	
DRB1*13				
ACPA-positive	16(8)	-	0.33(0.18–0.61)	3×10^−3^ (3×10^−2^)
ACPA-negative	18(19)	44(21)	0.89(0.48–1.63)	
DRB1*14				
ACPA-positive	34(17)	-	0.81(0.49–1.33)	
ACPA-negative	19(20)	43(20)	0.98(0.53–1.78)	
DRB1*15				
ACPA-positive	87(44)	-	1.28(0.86–1.90)	
ACPA-negative	43(45)	80(38)	1.35(0.83–2.20)	
DRB1*16				
ACPA-positive	3(2)	-	1.61(0.27–9.72)	
ACPA-negative	0(0)	2(1)	0.55(0.02–12.30)	

*The frequency of DRB1 genotypes in ACPA subgroups was calculated as the number of ACPA-positive or ACPA-negative RA cases for a specific DRB1 allele divided by the total number of cases within that ACPA subgroup (Indian: 200 ACPA-positive RA cases and 96 ACPA-negative RA cases).

§Individuals without the allele being investigated were used as the reference group for the calculation of odds ratio (OR) and 95% confidence interval (95% CI).

Corrected p (p_c_) values were obtained by multiplying the uncorrected p values by 13.

NS: not significant.

In Table 2 the frequencies of DRB1 alleles in the Malay controls were similar to that previously reported [21]. A significant increase in the frequency of DRB1*04 and DRB1*10 in cases with ACPA-positive RA was seen in comparison with controls. Although DRB1*16 tended to be positively associated with ACPA-positive RA, and DRB1*07 was negatively associated with ACPA-negative RA, these associations were not significant after correction for multiple comparisons. The frequencies of DRB1 alleles in the Chinese control subjects were comparable to that previously reported in the Singapore Chinese population [13]. The presence of DRB1*04 and DRB1*10 was significantly associated with an increased risk of ACPA-positive RA, whereas the presence of DRB1*03 and DRB1*14 alleles were significantly associated to a decreased risk of ACPA-positive RA. However, the effect of DRB1*03 was not statistically significant after correcting for multiple comparisons. DRB1*09, another reported RA-associated allele in Japanese and Korean populations, was common in both the patient and control groups for the Chinese population (Table 3). In Indians the DRB1*10 allele was significantly more frequent among ACPA-positive RA cases than among control subjects. Unlike the Malay and Chinese ACPA-positive RA, DRB1*04 allele represented insignificant risk regarding ACPA-positive RA among Indians. There was an increased frequency of DRB1*01 in ACPA-positive RA compared to the control group but this was not statistically significant. In addition, DRB1*13 may have had a protective role in the ACPA-positive RA among the Indian population (Table 4). We did not observe any DRB1 allelic group significantly associated with an increased risk of developing ACPA-negative RA in any of the three Asian ethnic groups.

### Protective effects conferred by different HLA-DRB1 alleles in relation to the presence or absence of shared epitope alleles

To assess the influence of shared epitope alleles on the association found for non-shared epitope alleles, we performed analyses of data with individuals who had neither the shared epitope nor the studied alleles as a reference group. In this study, we observed that DRB1*1202 allele was independent of the shared epitope in protection against disease development, both for ACPA-positive and ACPA-negative RA in the Malay population (p = 0.002 and p = 0.006, respectively). However, similar protective effect of DRB1*1202 allele was less significant for the Chinese ACPA-positive RA (p = 0.049) after the correction for shared epitope alleles influence. Interestingly, we also found that DRB1*1201 allele was significantly associated with susceptibility to ACPA-positive disease in the Malays, independent of the shared epitope alleles [OR 1.65 95% CI (1.13–2.42)] (Table 5).

**Table 5:** Association of DRB1*12 subtype alleles with the development of ACPA-positive or ACPA-negative RA among Malay and Chinese carriers with or without the shared epitope.

	Malay				Chinese			
	Cases (n = 405)	Controls (n = 685)	OR (95% CI)	P value	Cases (n = 210)	Controls (n = 164)	OR (95% CI)	P value
**ACPA-positive**								
No SE. no DRB1*12	65	291	Reference group		62	105	Reference group	
No SE. DRB1*1201	72	195	1.65 (1.13–2.42)	0.01	10	28	0.60 (0.27–1.33)	NS
No SE. DRB1*1202	7	113	0.28 (0.12–0.62)	0.002	2	14	0.24(0.05–1.10)	0.0489
SE. no DRB1*12	86	64	6.02 (3.95–9.16)	1.7×10^−18^	47	13	6.12(3.07–12.20)	4.3×10^−8^
SE. any DRB1*12	32	21	6.82 (3.70–12.59)	1.7×10^−11^	16	4	6.77(2.17–21.18)	0.0002
**ACPA-negative**								
No SE. no DRB1*12	72	291	Reference group		44	105	Reference group	
No SE. DRB1*1201	45	195	1.03 (0.68–1.57)	NS	17	28	1.44(0.72–2.91)	NS
No SE. DRB1*1202	9	113	0.36 (0.17–0.74)	0.006	1	14	0.17(0.02–1.33)	NS
SE. no DRB1*12	19	64	1.33 (0.75–2.37)	NS	10	13	1.83(0.75–4.49)	NS
SE. any DRB1*12	12	21	2.56 (1.19–5.46)	0.02	0	4	-	-

### Frequency of HLA-DRB1*04 subtypes

In order to investigate which DRB1*04 subtypes that associated with ACPA-positive RA in the three Asian populations, high resolution HLA-DRB1*04 subtype analysis was carried out in DRB1*04 positive patients and controls. Table 6 shows the frequency distribution of HLA-DRB1*04 subtypes among the DRB1*04 positive and ACPA-positive or ACPA-negative RA patients and controls.

**Table 6:** The frequency distribution of HLA-DRB1*04 subtypes among DRB1*04 positive and ACPA-positive or ACPA-negative RA patients by ethnic group.

	Malay€				Chinese¥				Indian§			
	Cases (n,%)	Controls (n,%)	OR, 95% CI	P value	Cases (n,%)	Controls (n,%)	OR, 95% CI	P value	Cases (n,%)	Controls (n,%)	OR, 95% CI	P value
DRB1*0401												
ACPA-positive	3 (3.1)	-			3 (3.1)	-			21 (30.4)	-		
ACPA-negative	0 (0)	2 (2.2)			1 (7.7)	2 (8.0)			4 (15.4)	12 (22.2)		
DRB1*0402												
ACPA-positive	1 (1.0)	-			0 (0)	-			1 (1.0)	-		
ACPA-negative	0 (0)	2 (2.2)			0 (0)	0 (0)			0 (0)	0 (0)		
DRB1*0403												
ACPA-positive	8 (8.2)	-	0.27, 0.11–0.65	0.0023	6 (9.5)	-	0.27, 0.08–0.89	0.0253	26 (37.7)	-	0.45, 0.22–0.93	0.0294
ACPA-negative	6 (24)	22 (24.4)			4 (3.1)	7 (28.0)			13 (50.0)	31 (57.4)		
DRB1*0404												
ACPA-positive	1 (1.0)	-			6 (9.5)	-			0 (0)	-		
ACPA-negative	2 (8.0)	7 (7.8)			1 (7.7)	1(4.0)			5 (19.2)	1(1.9)	12.6, 1.39–114.5	0.0208
DRB1*0405												
ACPA-positive	81 (82.7)	-	5.59, 2.91–10.76	7×10^−8^	46 (73.0)	-	03.83, 1.45–10.09	0.051	9 (13.0)	-		
ACPA-negative	10 (40.0)	46 (51.0)			4 (30.8)	10 (40.0)			2 (7.7)	4 (7.4)		
DRB1*0406												
ACPA-positive	2 (2.0)	-	0.15, 0.03–0.72	0.0162	6 (9.5)	-	0.15, 0.03–0.72	0.0162	0 (0)	-	0.15, 0.03–0.72	0.0162
ACPA-negative	1 (4.0)	11 (12.2)			3 (23.1)	4 (16.0)			1 (3.8)	1 (1.9)		
DRB1*0407												
ACPA-positive	0 (0)	-			0 (0)	-			1 (1.4)	-		
ACPA-negative	0 (0)	0 (0)			0 (0)	1 (4.0)			0 (0)	0 (0)		
DRB1*0408												
ACPA-positive	0 (0)	-			0 (0)	-			5 (7.2)	-		
ACPA-negative	0 (0)	0 (0)			0 (0)	0 (0)			0 (0)	2 (3.7)		
DRB1*0410												
ACPA-positive	2 (2.0)	-			0 (0)	-			6 (8.7)	-		
ACPA-negative	0 (0)	0 (0)			0 (0)	0 (0)			1 (3.8)	3 (5.6)		
DRB1*0401/*0404/*0405/*0408/*0410												
ACPA-positive	87 (88.8)	-	1.94, 2.36–10.73	1×10^−5^	52 (81.3)	-	13.72, 4.51–41.72	3×10^−7^	41 (59.4)	-	2.13, 1.03–4.4	0.0397
ACPA-negative	18 (72.0)	55 (61.1)	1.64, 0.62–4.32	0.3171	6 (46.2)	13 (52.0)	2.71, 0.65–11.29	0.1663	12 (46.2)	22 (40.7)	1.25, 0.49–3.20	0.6464

€,¥,§The frequency of DRB1 genotypes in ACPA subgroups was calculated as the number of ACPA-positive or ACPA-negative rheumatoid arthritis(RA) cases for a specific DRB1 allele (DRB1*04 +)divided by the total number of cases within that ACPA subgroup (Malay :98 DRB1*04+/ACPA-positive cases, 25 DRB1*04+/ACPA-negative cases; Chinese: 64 DRB1*04+/ACPA-positive cases, 13 DRB1*04+/ACPA-negative cases; Indian: 69 DRB1*04+/ACPA-positive cases, 26 DRB1*04+/ACPA-negative cases).

The frequencies of DRB1*0405 were largely increased in the Malay and Chinese ACPA-positive RA cases as compared to their control groups. The Indian ACPA-positive RA cases displayed a much more limited increased frequency of DRB1*0405 in comparison with the controls and this difference was not statistically significant. DRB1*0403 was significantly associated with protection against ACPA-positive RA in all the three ethnic groups. The predominance of DRB1*0403 in Indian patients and controls resulted in a concomitant decrease in all other DRB1*04 subtype alleles including DRB1*0405. Further analysis of the DRB1*04 subtype SE alleles (DRB1*0401, DRB1*0404, DRB1*0405, DRB1*0408 and DRB1*0410) in the context of ACPA subgroups revealed a significant association between DRB1*04 SE alleles and ACPA-positive RA in all the three ethnic groups. No DRB1*04 subtype conferred any significantly increased risk of developing ACPA-negative RA in the Malaysian populations. However, a trend towards an association between DRB1*0404, and ACPA-negative RA was seen in the Indian population but the number of observations was quite small.

### Effect of single or double HLA-DRB1-shared epitope (SE) alleles

As is seen from Table 7, presence of two SE alleles (double SE alleles) conferred a higher risk than presence of single SE allele in all three populations. Also in this analysis, we failed to observe any significant risk of developing ACPA-negative RA in relation to the presence of SE alleles. Furthermore, the presence of SE alleles was a risk factor for IgM and IgG RF-positive RA in all three populations (for IgM-positive RF, Malays, OR 5.82 [4.06–8.34]; Chinese OR 5.72 [3.02–10.83]; Indians OR 2.83[1.86–4.32]; for IgG-positive RF, Malays, OR 5.55 [3.89–7.92]; Chinese OR 5.32 [2.81–10.07] and Indians OR 2.69[1.77–4.10]) as well as IgM and IgG RF-negative RA in the Malay and Chinese populations, but not in the Indian population (for IgM-negative RF, Malays, OR 2.52 [1.73–3.68]; Chinese OR 3.69 [1.93–7.07]; Indians OR 1.53[1.00–1.67]; for IgG-negative RF, Malays, OR 2.58 [1.76–3.78]; Chinese OR 3.96 [2.07–7.59] and Indians OR 1.62[1.04–2.54]). Because the majority of RF-positive/negative patients with RA were also ACPA-positive, associations of RF-positive/negative RA may be a consequence of the underlying ACPA status. The presence of two SE alleles (double SE alleles) was also conferred a higher risk than the presence of single SE allele for both IgM and IgG RF in all three populations (data not shown).

**Table 7:** Risk of developing ACPA-positive or ACPA-negative RA among carriers of SE (single, double or any) in the MyEIRA study population by ethnicity.

	No SE*		Single SE			Double SE			Any SE		
	Cases	Controls	Cases	Controls	OR (95% CI)	Cases	Controls	OR (95% CI)	Cases	Controls	OR (95% CI)
Malay											
ACPA-positive	140	600	100	79	5.29 (3.74–7.48)	16	4	17.14(5.64–52.07)	116	83	5.85(4.18–8.18)
ACPA-negative	119		30		1.87 (1.18–2.97)	0		1.78 (1.12–2.82)	30		1.82(1.15–2.89)
Chinese											
ACPA-positive	72	147	54	17	6.49 (3.51–11.98)	6	0	0.00(-)**	60	17	7.20 (3.92–13.23)
ACPA-negative	62		9		1.26 (0.53–2.97)	1		0.00(-)#	10		4.74 (0.16–143.20)
Indian											
ACPA-positive	88	148	98	56	2.75 (1.81–4.16)	14	5	4.71 (1.64–13.52	112	61	2.89 (1.93–4.34)
ACPA-negative	65		29		1.10 (0.65–1.87)	2		1.09 (0.65–1.82)	31		0.91 (0.17–4.82)

*Cases and controls without SE genes were used as reference groups.

**Fisher exact two-tailed p value, p = 0.0015.

#Fisher exact two-tailed p value, p = 0.3, NS.

## Discussion

The major finding in this study is that different DRB1 shared epitope (SE) alleles, that are common in Asian but not Caucasian populations confer a significant risk of developing ACPA-positive RA, but not ACPA-negative RA in all three Asian populations from Malaysia.

Previous studies have demonstrated a significant association between the presence of SE alleles and risk for ACPA-positive RA mainly in Caucasian, but also in some non-Caucasian populations [22], [23], [24], [25]. One study from Korea has, however, observed that ACPA antibodies in Koreans are strongly associated with RA susceptibility, independent of SE status, suggesting that the association between HLA-DRB1 SE and ACPA-positive RA might be dependent on ethnicity-dependent genetic variations. On the other hand, the association between SE alleles (DRB1*0401, *0404, *0405, *0410) and ACPA status was not found in the Chinese Han RA patients by Xue et al [10], [20]. However, the discrepancy observed between Xue et al and our study for the Chinese population could be explained by the relatively small samples size (RA patients, n = 81) and by the fact that not all SE alleles (e.g not DRB1*01 and DRB1*10) were considered in the analysis [10], [20]. The results of our present study however demonstrate that SE-positivity is highly associated with ACPA-positive RA for all three ethnic groups in Asia, thus providing support for the notion that this association is present also in some non-Caucasian populations.

The percentage of RA patients possesing the SE alleles in current study was comparable to those of other Asian populations [10], [11]. but was lower in comparison with Caucasians RA patients (81.6% in Northern Irish and 87.7% in Southern Swedish) [26], [27]. On the other hand, the frequency of SE alleles in the European healthy controls was also relatively high (54.9% in Northern Irish and 50% in Southern Swedish), when compared with various healthy controls in the Asian populations [9], [10], [11], [13], [26], [27]. Notably, out of the three ethnic groups in our study, Indian RA patients and controls had a slightly higher frequency of SE (56% in Indians RA vs. 28.6% in Indian controls), though it was still lower than in European Caucasian populations [26], [27]. Despite the lower frequency of SE alleles in RA patients and control subjects in our study, the estimated relative risk for SE-positive individuals to develop ACPA-positive RA was comparable to that noted for European Caucasians (OR 7.65 in Southern Swedish [27] and OR 3.91 in Northern Irish [26]). This suggests that similar genetic mechanisms may operate in several different ethnic groups in HLA-DRB1-dependent immune activation for RA pathogenesis.

HLA-DRB1*0401 and *0404 alleles are the most common SE-encoding DRB1*04 subtypes associated with the ACPA-positive RA in Caucasians [25], [27]. These alleles were however, rare in our population, while DRB1*10 was the most prevalent SE-encoding allele in all three ethnic groups. Interestingly, the DRB1*10 specificity is associated with RA also among the Eastern European [23] and Southern European populations [28], but it is seldom found in Caucasians of the North European ancestries [25], [27].

Another feature of the genetic background in our study revealed a relatively low frequency of DRB1*01, another SE-encoding DRB1 allelic group in the Malaysian RA populations. This is parallelled by the HLA-DRB1 allele distribution seen in the control subjects (approx. 2% in this study). Nevertheless, when the DRB1*01 allele was observed more frequent in RA patients, it demonstrated a more clear trend towards association, e.g in Indian ACPA-positive RA patients. DRB1*01 is present at comparable frequencies in various Caucasian populations including 20% in Swedish, 23.3% in Spanish, and 25.3% in Northern Irish [26], [27], [28]. We could not assess the risk effect between DRB1*01 and RA development for the three Asian ethnicities due to relatively small number of observations in our study population.

Further analysis demonstrated a negative association between DRB1*1202 allele and ACPA-positive RA in the Malay and Chinese populations, suggesting a protective role of this allele. After the correction for shared epitope alleles, DRB1*1202 which encodes the motif ^70^DRRAA^74^ was still significantly associated with protection regarding ACPA-positive RA in these ethnic groups. DRB1*1202 has been described by a few studies [11], [13], [16], [19], [22], [29]. However, only one study showed a protective effect of DRB1*1202 in RA, but this effects was non-significant and the study did not analyse ACPA-positive and –negative RA subsets separately [11]. The DRB1*13 allele has been found significantly decreased in RA patients compared to controls in other studies [2], [19], [25], [30], [31]. In the present study, theDRB1*13 allele was significantly decreased among the ACPA-positive RA cases compared with the controls in the Indian population, suggesting a protective role of this allele regarding ACPA-positive RA, though the sample set examined was relatively small and the result need further confirmation.

In conclusion, our study describes the association between ACPA-positive RA and certain HLA-DRB1 SE alleles that are common in Asian but not in Caucasian populations, and it also describes protective effects of some HLA-DRB1 alleles in an ethnicity-dependent manner. These findings provide a novel basis for analysis of the relationship between specific HLA-DRB1 subtypes and specific immune reactions of potential importance for the pathogenesis of RA. The possibility to analyze these relationships further in different ethnic groups will be of major importance for our understanding of which immune reactions that may contribute to RA in different subsets in different parts of the world, and to learn which genes and which mechanisms that promote or counteract the pathogenic processes.

## Materials and Methods

### Study design

This is a multicenter case-control study entitled the Malaysian Epidemiological Investigation of Rheumatoid Arthritis (MyEIRA) similar to the Swedish EIRA study [32], [33]. It involves incident cases of rheumatoid arthritis (RA), derived from the population in the Malay Peninsula. The recruitment period for the cases and controls was carried out from July 2005 to December 2009.

### Patients and control subjects

A total of 1,079 patients with RA and 1,470 healthy controls were enrolled in the study. Of the 1,079 cases, 450 (41.7%) were Malays, 223 (20.7%) were Chinese, 331 (30.7%) were Indians and 75 (7.0%) were of mixed ethnicities. Each case was defined as a person who fulfilled the American College of Rheumatology (ACR) 1987 revised criteria for the classification of RA [34]. All potential cases were examined and diagnosed by rheumatologists. Eight government hospital rheumatology units participated in this study. Of the RA patients, 863 (86%) were females and 141 (14%) were males. The median disease duration for RA was one year (Inter Quartile Range, IQR = 2 years). Radiological findings were recorded as absence or presence of hand joint erosions. Radiographic erosion data was available for 974 RA patients (90.3%). Out of these, 169 of 974 (17.4%) had erosive RA. Extra-articular manifestations were noted in 11.7% of 974 RA patients: sicca syndrome, n = 52 (5.3%), pulmonary fibrosis, n = 28 (2.9%), rheumatoid nodules, n = 19 (2.0%), secondary sjögren, n = 14 (1.4%) and vasculitis, n = 1 (0.1%). 50.3% and 50.8% of the RA patients were positive for IgM and IgG RF, respectively. For each case, a control subject was randomly selected taking into consideration the subject's age, sex and residential area. Controls with a history of autoimmune disease were excluded. In this study, the control subjects were recruited based on two methods: (i) hospital-based healthy controls, which comprised mainly nurses and allied health care workers from the same hospital as the patients; and (ii) population-based healthy controls, residing in the same geographic location as the patient. Of the 1,470 control subjects, 448 or 30.5% were hospital-based controls, and 1,022 population-based controls. Of the control subjects, 1,213 (87.8%) were females and 169 (12.2%) were males. All patients and controls were unrelated and the ethnic background was assessed by self-description based on questions on ancestry. For example, an individual is defined as Malay ethnic group when both he/her parents are Malays. Interracial marriages are fairly common among the Malaysians. Therefore, individuals of interracial marriage parents were categorized as others/mixed ethnicities and they were excluded for the analysis. In our study, we restricted only patients and controls with both parents from the same ethnic group were included leaving 1,004 RA patients and 1,382 control subjects for the analysis.

The baseline demographic characteristics of patients and controls are shown in Table 1. Malays are an ethnic group of Austronesian people predominantly inhabiting the Malay Pennisula. In Malaysia, the Malays forms the dominance race and make up to 50% of the total population. The Chinese population consists mainly of descendents of Han Chinese settlers from Southern China, particularly the provinces of Fujian, Guandong and Hainan. They represent the second largest ethnic group in Malaysia, accounting 24% of the total population. The Indian population is largely descendents from those who migrated from Southern Indian during the British colonization of Malaya. The Indian population is the third largest ethnic group (7% of the total population) in Malay Pennisula. More than ninety percent of the Indian migrants were ethnic Tamil, Telugus, Malayalees and the remainder of the Indians comprising Punjabis, Bengalis, Gujaratis and Sindhis from Northern India. All the participants were informed about the research, and written consent was obtained. The study was approved by the Medical Research and Ethics Committee, Ministry of Health, Malaysia.

### DNA extraction

White cells were separated from 20 ml of EDTA blood using Ficoll Hypaque (Lymphoprep™, Axis-Shield PoC AS, Oslo, Norway) and the DNA was extracted using the QIAamp DNA Blood Mini kit from Qiagen (Hilden, Germany). All DNA was stored at −20°C until tested.

### HLA-DRB1 genotyping

Low to medium-resolution and high-resolution genotyping of HLA-DRB1 alleles were performed by the polymerase chain reaction and sequence-specific oligonucleotide probe hybridization method using the LABType® SSO Class II DRB1 and LABType® HD Class II DRB1 (One Lambda Inc., CA, USA), respectively, with Luminex Multi-Analyte Profiling System (xMAP, Luminex Corporation, Texas, USA), according to the manufacturer's instruction. The assignment of HLA typing was accomplished using the HLA Fusion software (version 1.3.0) provided by the manufacturer (One Lambda Inc., CA, USA). Among the HLA DRB1 alleles, DRB1*01, DRB1*0401, DRB1*0404, DRB1*0405, DRB1*0408, DRB1*0410 and DRB1*10 were defined as SE alleles [25]. Any genotype with combination of two of these alleles was considered to be double SE genotypes.

### Autoantibody Measurements

ACPA were identified and quantified with Immunoscan-RA Mark2 ELISA test (anti-CCP test, Malmö, Sweden). Samples with results ≥25 AU/mL were defined as positive. IgM and IgG rheumatoid factor (RF) was determined by ELISA kits (Immuno-Biological Laboratories, Hamburg, Germany). Samples with results >15 IU/ml were defined as positive.

### Statistical analysis

Allele frequencies were obtained by direct counting. When only one allele was detected in genotyping assay, the individual was considered homozygous and the allele was counted twice. The frequencies and odds ratios (ORs) with 95% confidence intervals (95% CIs) of the alleles and genotypes of DRB1 were compared between patients and control subjects using the Chi-square or Fisher's exact test, where appropriate. Corrected p (P_c_) was obtained by multiplying the observed p values by the number of allelic group examined: 13 for HLA-DRB1. A probability of 0.05 (2 tailed) was used as a significance threshold. Student t-test was performed for the mean age of the patients and control subjects group. Standard software (SPSS 17.0 for Windows, SPSS Chicago, IL, USA) was used for statistical analyses.
